# LncRNA LINC00641 Sponges miR-497-5p to Ameliorate Neural Injury Induced by Anesthesia via Up-Regulating BDNF

**DOI:** 10.3389/fnmol.2020.00095

**Published:** 2020-06-30

**Authors:** Qingxia Chen, Jingjia Yan, Wenji Xie, Wenqin Xie, Meijun Li, Yanle Ye

**Affiliations:** ^1^Department of Anesthesiology, The First Affiliated Hospital of Fujian Medical University, Quanzhou, China; ^2^Department of Nursing, Quanzhou Medical College, Quanzhou, China; ^3^Department of Urology, The First Hospital of Quanzhou, Quanzhou, China

**Keywords:** ketamine, neural injury, LINC00641, miR-497-5p, BDNF

## Abstract

**Introduction:**

Ketamine, which is widely used in anesthesia, can induce cortical neurotoxicity in patients. This study aims to investigate the effects of long non-coding RNA LINC00641 on the ketamine-induced neural injury.

**Materials and Methods:**

In this study, rat pheochromocytoma cells (PC12 cells) were used as a cell model and Sprague–Dawley postnatal day 7 rats were used for experiments *in vivo*. Ketamine-induced aberrant expression levels of LINC00641, miR-497-5p and brain-derived neurotrophic factor (BDNF) were examined by qRT-PCR. The effects of LINC00641 and miR-497-5p on ketamine-induced neural injury were then examined by MTT assays and TUNEL analysis. In addition, the activity of ROS and caspase-3 was measured. The regulatory relationships between LINC00641 and miR-497-5p, miR-497-5p and BDNF were detected by dual-luciferase reporter assay, respectively.

**Results:**

Ketamine induced the apoptosis of PC12 cells, accompanied by down-regulation of LINC00641 and BDNF, and up-regulation of miR-497-5p. LINC00641 overexpression enhanced the resistance to the apoptosis of PC12 cells, while transfection of miR-497-5p had opposite effects. Furthermore, LINC00641 could bind to miR-497-5p and reduce its expression, but indirectly increase the BDNF expression, which was considered as a protective factor in neural injury and activated TrkB/PI3K/Akt pathway.

**Conclusion:**

Collectively, LINC00641/miR-497-5p/BDNF axis was validated to be an important signaling pathway in modulating ketamine-induced neural injury.

## Introduction

Ketamine, also known as special K, is a non-competitive *N*-methyl-D-aspartate (NMDA) receptor antagonist. Due to its good safety and sedative analgesic effects, ketamine is widely used in diverse fields, such as pediatric surgical anesthesia, chronic pain treatment and so on (Marland et al., 2013; Pourmand et al., 2017). Nevertheless, previous studies have shown that ketamine can induce dose-dependent neurotoxicity *in vivo* and *in vitro*, especially during brain development (Pang et al., 2018). Moreover, studies have revealed that neuronal apoptosis is one of the common mechanisms of anesthesia-induced neurotoxicity development (Li et al., 2013). Ketamine-induced neuronal cell death involves compensatory upregulation of NMDA receptors, and up-regulation of NMDA receptors leads to calcium overload, which exceeds mitochondrial buffering capacity and results in a degree of disorder that interferes with electron transport, thereby leading to the production and subsequent neuronal injury, apoptosis of reactive oxygen species (ROS) (Liu et al., 2013). However, the molecular mechanism by which ketamine induces neuronal apoptosis remains largely unknown.

Long non-coding RNAs (lncRNAs) are non-coding RNAs (ncRNAs) greater than 200 nucleotides in length (Bar et al., 2016). lncRNAs are engaged in a wide range of biological and cellular processes (Alahari et al., 2016). What’s more, studies have also revealed that lncRNAs are engaged in the development of the nervous system and the regeneration process after neurological injury (Wu et al., 2013). LINC00641 is one member of lncRNAs, which has been found to play a role in modulating the proliferation and apoptosis of non-small cell lung cancer cells (NSCLC) (Li et al., 2019). Nonetheless, the role of LINC00641 in ketamine-induced neuronal apoptosis has not been reported.

MicroRNAs (miRNAs) also belong to ncRNAs with about 22 nucleotides in length. Functionally, miRNAs interact with complementary sequences of the targeted messenger RNA (mRNA) in the 3′ untranslated regions (3′ UTR) sites after transcription, thus promoting the degradation of mRNA and inhibiting protein expression. Studies have revealed that miRNAs are abundantly expressed in the brains of diverse species and play a key role in mediating nerve and oligodendrocyte development (Lau and Hudson, 2010; Zhao et al., 2010). For instance, miR-34c modulates the PKC-ERK pathway and plays an essential role in ketamine-induced apoptosis in hippocampal neurons (Cao et al., 2015). Additionally, miR-497-5p promotes ischemic neuronal death by negatively regulating anti-apoptotic proteins (Li et al., 2015; Sinoy et al., 2017). However, the regulation mechanism of miR-497-5p in ketamine-induced neuronal apoptosis has not been explored.

Brain-derived neurotrophic factor (BDNF) has been confirmed to be a key regulator of neurite outgrowth, synaptic plasticity, and functional neuronal junction selection in the central nervous system (Lewin and Barde, 1996). BDNF binds to tropomyosin receptor kinase B (TrkB) to stimulate and facilitate the growth and differentiation of nerve cells, preventing neuronal injury and death (Chao, 2003). Studies have demonstrated that ketamine mediated neuronal injury leads to the down-regulation of BDNF (Zuo et al., 2016). In this article, our data revealed that under the effect of higher concentration of ketamine, neuronal apoptosis was significantly increased, accompanied by a remarkable decrease in the LINC00641 and BDNF expression, and a significant up-regulation of miR-497-5p. Therefore, we speculate that high concentration of ketamine can attenuate the LINC00641 expression, and then indirectly down-regulate the BDNF expression through miR-497-5p, resulting in neuronal apoptosis.

## Materials and Methods

### Animals and Drug Treatment

Sprague–Dawley postnatal day 7 (PND7, 14–18 g) rat pups were used in all *in vivo* assays. The rats were treated and manipulated according to the National Institutes of Health guide for the care and use of laboratory animals (NIH Publications No. 8023, revised 1978). All operations minimized animal suffering and reduced the number of animals used. First, rats were randomly divided into ketamine and control (saline) groups. Ketamine group: rats were intraperitoneally injected with 0, 10, 20, and 30 mg/kg ketamine for three consecutive days. Control group: rats were injected with 0.9% saline for three consecutive days. Twenty-four hours after the last administration, the rats were anesthetized and sacrificed to collect hippocampus tissue and stored in the refrigerator at −80°C. In addition, the PI3K inhibitor LY-294002 (1 mg/kg) was injected intraperitoneally before ketamine treatment to determine whether the protective effect of LINC00641 depended on the PI3K/Akt pathway.

### Stereotaxic Surgery

Rats underwent stereotactic surgery with a stereotaxic frame David Kopf Instrument, Tujunga, CA; Model 963 Ultra Precise Small Animal Stereotaxic Instrument, supplemented with 2.5 μL Hamilton syringe (Hamilton Medical, Reno, NV, United States; 87942) and bilateral microsyringe pump controller [UltraMicroPump (UMP3) with SYS-Micro4 Controller – World Precision Instruments, Sarasota, FL, United States; UMP3-1] to inject hippocampus with rAAVs, which carried the LINC00641 overexpressing vector, serotype 2/9 (Penn VectorCore, University of Pennsylvenia, United States). Injection coordinates were: before and after: +2.1 mm; medial-lateral: ±1.5 mm; dorsal-ventral: −1.8 mm. The total volume of injection was 0.75 μL of viral vector solution at an injection rate of 0.25 μL/min.

### Pheochromocytoma Cell Culture and Processing

Pheochromocytoma cells (PC12) were purchased from the American Type Culture Collection (CRL-1721, ATCC, Manassas, VA, United States) and cultured in cell culture dishes (5 × 10^5^ cells/dish) or 96-well tissue culture dishes (1 × 10^4^ cell/well) with neuron differentiation medium in a humidified incubator containing 5% CO_2_ at 37°C. The medium was changed every 48 h. After the monolayer cultured cells were confluent, the cells were trypsinized with 0.25% trypsin and subcultured, and the cells in the logarithmic growth phase were taken for assays. Two weeks later, PC12 neuronal cells were treated with different concentrations of ketamine (0, 10, 50, or 100 μM) for 24 h. Subsequently, cell viability (MTT analysis), apoptosis assay (quantification of caspase-3 activity), and ROS levels were measured immediately after exposure to ketamine for 24 h.

### Cell Transfection

MiR-497-5p mimics, miR-497-5p inhibitors and corresponding NC inhibitors (NC mimic and NC inhibitor) were synthesized by GenePharma (Shanghai, China). The entire coding sequence of LINC00641 was cloned into the pcDNA3.1 plasmid (Sangon Biotech, Shanghai, China) to construct the overexpression vector pc-LINC00641. Short-hairpin RNAs (shRNAs) against LINC00641 were ligated into the pGPU6/Neo plasmid (GenePharma) to inhibit the LINC00641 expression, named sh-LINC00641. Empty vectors pcDNA3.1 and shRNA scramble were used as blank controls. The cells were transfected with Lipofectamine 3000 reagent (Life Technologies Corporation, Carlsbad, CA, United States) in a 6-well plate (5 × 10^5^ cells/well) for 48 h. The LINC00641-overexpressed cell line was also treated with TrkB inhibitor (K-252a, 1.7 nM, MCE, United States) for 6 h.

### Measurement of Cell Viability

MTT was converted to formazan crystals using mitochondrial dehydrogenase, and cell viability were determined by MTT assay. According to the experimental design, the cells supplemented with 10 μL of 5 mg/mL MTT solution was incubated at 37°C for 4 h. The medium containing MTT was removed, and 200 mL of dimethyl sulfoxide (DMSO) was added to each well to dissolve formazan crystals. The absorbance was measured at 492 nm using a microtiter plate reader (ThermoFisher, Shanghai, China). The absorbance of the control group (A control) was set to a survival rate of 100%. The absorbance of the treated cells (A experimental) correlated with the absorbance of the control cells and was normalized. The background was the absorption rate of the medium plus MTT in the absence of cells. Cell survival rate was defined as cell viability = [(A experimental − A background)/(A control − A background)] × 100%.

### TUNEL Assay

Apoptosis-related DNA fragmentation was analyzed by terminal deoxynucleotidyl transferase-mediated deoxynucleotidyl triphosphate *in situ* nick-end labeling (TUNEL) staining using *in situ* apoptotic cell death detection kit (Roche Applied BioSciences) according to the manufacturer’s instructions. The cells cultured on the coverslips were washed with PBS and fixed with ice-cold 1% paraformaldehyde. Subsequently, the terminal deoxynucleotidyl transferase (a template-independent polymerase) was employed to incorporate nucleotides at the DNA cleavage site. Following that, nuclei were stained with TO-PRO^®^-3 and fluorescence images were taken on three different fields of view of each coverslip using a confocal microscope. The apoptotic index was calculated as a percentage according to the following formula: TUNEL positive cell number/total cell nucleus.

### Reactive Oxygen Species Measurement

After ketamine treatment, PC12 neuronal cells were re-implanted into 96-well plates and detected using the Cellular Reactive Oxygen Species Detection Assay Kit (Abcam, United States) according to the manufacturer’s protocol. After that, the 96-well plate was moved to a Multiscan plate-reader (Thermo Fisher Scientific, United States), and the activity of the ROS was quantified using the absorbance measured at 530 nm according to the manufacturer’s protocol.

### Caspase-3 Activity Assay

The activity of caspase-3 was detected by the caspase-3 colorimetric assay kit. Briefly, 6 h after the last injection, the young rats were sacrificed by decapitation, and the brain tissue was quickly separated. Fresh frozen brain tissue was homogenized in lytic buffer to extract protein or extract protein directly from PC12 neural cell line. The protein concentration was then determined using the Bradford Protein Assay Kit. After that, 50 μL of the lysate supernatant containing 200 μg of protein, 50 μL of reaction buffer, and 5 μL of caspase-3 substrate was incubated in a 96-well plate at 37°C for 4 h with a microtiter plate reader (ThermoFisher, Shanghai, China). The absorbance was measured at a wavelength of 400 nm. The absorbance of the blank sample without the caspase-3 substrate was subtracted from these values. The results implied that the final caspase-3 activity was the ratio of the control group.

### Quantitative Polymerase Chain Reaction Assay

Total RNA was extracted from the cells using TRIzol reagent (Invitrogen, United States) according to the manufacturer’s instructions. The purity and concentration of RNA were determined using a UV spectrophotometer and the integrity of the RNA was verified by agarose gel electrophoresis. According to the manufacturer’s instructions, Primescript^TM^ RT reagent kit with gDNA Eraser (TaKaRa, Japan) was employed to synthesize cDNA templates. The qPCR analysis was quantified mature miRNA expression with the NCode miRNA qRT-PCR analysis (Invitrogen), or mRNA expression with SYBR Green PCR Master Mix (TaKaRa, Ohsu, Japan). The relative gene expression values were calculated by comparing the 2^–ΔΔct^ method. The mRNA took GAPDH as an internal reference and the miRNA took U6-snRNA as an internal reference. The PCR primer for BDNF was: forward primer: 5′-GGGACCGGTTTGTGT-3′, reverse primer: 5′-TTGCTTTTTCATGGGGGCA-3′. LINC00641 forward, 5′-G TAACTCTATGTACAACGTTAA-3′, reverse primer: 5′-TA GAAGTCAACTCATTATGCTGCTG-3′; PCR primer for GAP DH: forward primer: 5′-GACAGCCGCATCTTCT-3′, reverse primer: 5′-GCGCCCAATACGACCAAATC-3′; miR-497-5p forward primer: 5′-CAGCAGCACACTGTGGTTTGTA-3′, reverse primer was universal primers (Uni-miR qPCR Primer), U6-snRNA, forward primer: 5′-CTCGCTTCGGCAGCACA-3′, reverse primer: 5′-AACGCTTCACGAATTTGCGT-3′.

### Dual-Luciferase Reporter Assay

The cDNA sequences of wild type (WT) BDNF and LINC00641 3′-UTR were cloned into pGL3 vectors (Promega, Madison, MI, United States), and a dual-luciferase reporter vector BDNF-WT and LINC00641-WT were established. To generate the mutated 3′-UTR, the mutation was generated using the QuikChange II XL Site-Directed Mutagenesis Kit (Stratagene). The mutated BDNF and LINC00641 3′-UTR sequences were also cloned into pGL3 vectors to establish another dual-luciferase reporter vector BDNF-Mut and LINC00641-Mut. In addition, one synthetic miR-497-5p mimics and one non-specific NC mimics were procured from GenePharma (Shanghai GenePharma, Shanghai, China). PC12 cells were incubated in a 48-well plate (2 × 10^3^ cells/well). After 24 h of incubation, Lipofectamine 2000 (Invitrogen) was employed to transfect indicator cells according to the manufacturer’s instructions. After 48 h, the dual-luciferase reporter assay system (Promega) was employed to measure firefly luciferase activity. All experiments were performed in triplicate and repeated three times independently.

### Western Blot

Total protein was extracted from cultured cells or fresh frozen rat brain tissue, and protein concentration was determined using the BCA protein assay (Beyotime Institute of Biotechnology, China). Twenty micrograms of protein from each sample were separated by 12 or 15% SDS-PAGE and electrotransferred onto a PVDF membrane (Millipore, United States) for immunoblot analysis. Rabbit anti-p-TrkB (Tyr515) (1:1,000, ab131483; Abcam, Cambridge, United Kingdom), rabbit anti-TrkB (1:2,000, ab33655; Abcam), PI3K (1:1,000, ab125633; Abcam), p-PI3K (Tyr607) (1:500; ab182651; Abcam), rabbit anti-Akt (1:1,000; ab126580; Abcam), rabbit-anti-p-Akt (Ser473) (1:1,000; ab38449; Abcam), anti-GSK-3β (1:5,000; ab32391, Abcam), ant-p-GSK-3β (Tyr216) (1:1,000; ab75745; Abcam), anti-CREB (1:1,000, ab31387; Abcam), anti-p-CREB (Ser133) (1:1,000, ab32096; Abcam), anti- BDNF (1:1,000, ab108319; Abcam), anti-Bax (1:1,000; ab32503 Abcam), anti-Bcl-2 (1:1,000; ab59348; Abcam), and anti-β-actin (1:1,000, ab6276, Abcam) and other primary antibodies were incubated on the membrane. After being shaken and observed at 4°C, horseradish peroxidase-conjugated goat anti-rabbit IgG (1:5,000, BL003A; Biosharp, St. Louis, MO, United States) was incubated for 2 h at room temperature and the bands were detected by enhanced chemiluminescence for visualization (Thermo Scientific, Waltham, MA, United States) and analyzed in ImageJ software. In the statistical analysis, all target proteins were normalized to β-actin on the same membrane and then calculated as the percentage of the control group.

### ELISA

Quantitative analysis of BDNF in samples was performed using the BDNF Emax^®^ ImmunoAssay System (Promega, Madison, WI, United States) according to the manufacturer’s instructions. The optical density (OD) at 450 nm was measured using the microplate reader. The samples were analyzed in triplicate with the average value.

### RNA Pull-Down Assay

LINC00641 and miR-497-5p binding site mutation LINC00641 (LINC00641-mut) was transcribed *in vitro* from pSPT19-LINC00641 and pSPT19-LINC00641-mut, respectively. SP6 RNA polymerase (Roche) and biotinylated biotin RNA were employed to label mixture (Roche) according to the manufacturer’s instructions. After treatment with RNase-free DNase I (Roche), the biotinylated RNA transcribed *in vitro* was purified by RNeasy Mini Kit (Qiagen, Valencia, CA, United States). After that, 3 μg of purified biotinylated RNA was incubated with 1 mg whole cell lysate from PC12 cells for 1 h at 25°C and then recovered with streptavidin agarose beads (Invitrogen). The RNA existed in the pull-down material was quantified by qRT-PCR as previously described.

### Statistical Analysis

Data were expressed as the mean ± SD of at least three independent assays in each cell experimental group. All statistical analyses were performed using one-way analysis of variance (ANOVA) followed by the post hoc Duncan test. The analysis was performed using GraphPad Prism (version 5; GraphPad Software, Inc., La Jolla, CA, United States). *P* < 0.05 was considered statistically significant.

## Results

### High Concentration of Ketamine-Induced Neuronal Apoptosis and Down-Regulation of LINC00641 in Rat Brain

**FIGURE 1:**
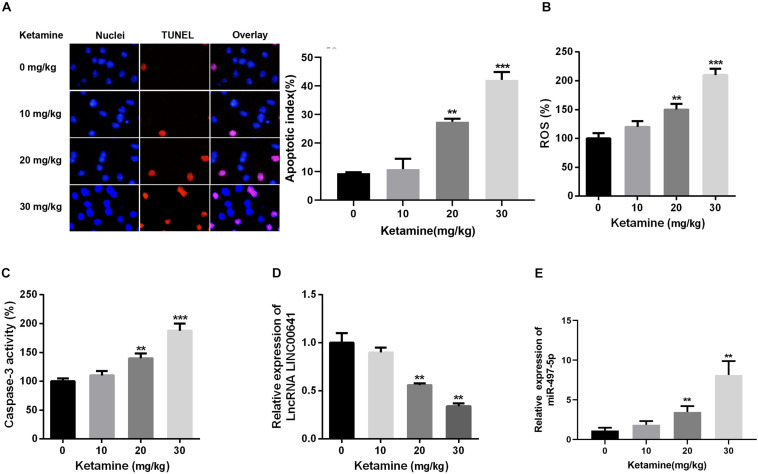
High-concentration of ketamine-induced neuron apoptosis and down-regulation of LINC00641 *in vivo*. Rat hippocampus treated with different concentrations of ketamine (0, 10, 50, and 100 mg/kg). **(A)** TUNEL was employed to analyze the neuronal apoptosis and apoptotic index, the nuclei were blue, the apoptotic cells were red, and the apoptotic index was calculated by TUNEL positive neurons/total neuron number. **(B)** ROS levels in rat brain were determined under treatment of different concentrations of ketamine. **(C)** The activity of caspase-3 in rat brain was measured under the treatment of different concentrations of ketamine. **(D,E)** The relative expression of LINC00641 and miR-497-5p in rat brain treated with different concentrations of ketamine was analyzed by qRT-PCR. **P < 0.01 and ***P < 0.001 compared with 0 mg/kg group.

In this article, the rat brain was injected with 0, 10, 20, and 30 mg/kg ketamine. The apoptosis of brain nerves was detected by TUNEL staining. The results revealed that the number of apoptotic cells in the high-concentration ketamine-treated group was significantly increased (Figure 1A). Additionally, as the concentration of ketamine increased, ROS levels and caspase-3 activity also increased significantly (Figures 1B,C). What’s more, we evaluated the LINC00641 and miR-497-5p expression by qPCR. The results implied that the LINC00641 expression was remarkably decreased, while the miR-497-5p increased in brain tissue in comparison with the control group (Figures 1D,E). These results indicated that LINC00641 expression might participate in ketamine-induced neuronal injury.

### The Effect of LINC00641 on Ketamine-Induced Neuronal Injury *in vitro*

**FIGURE 2:**
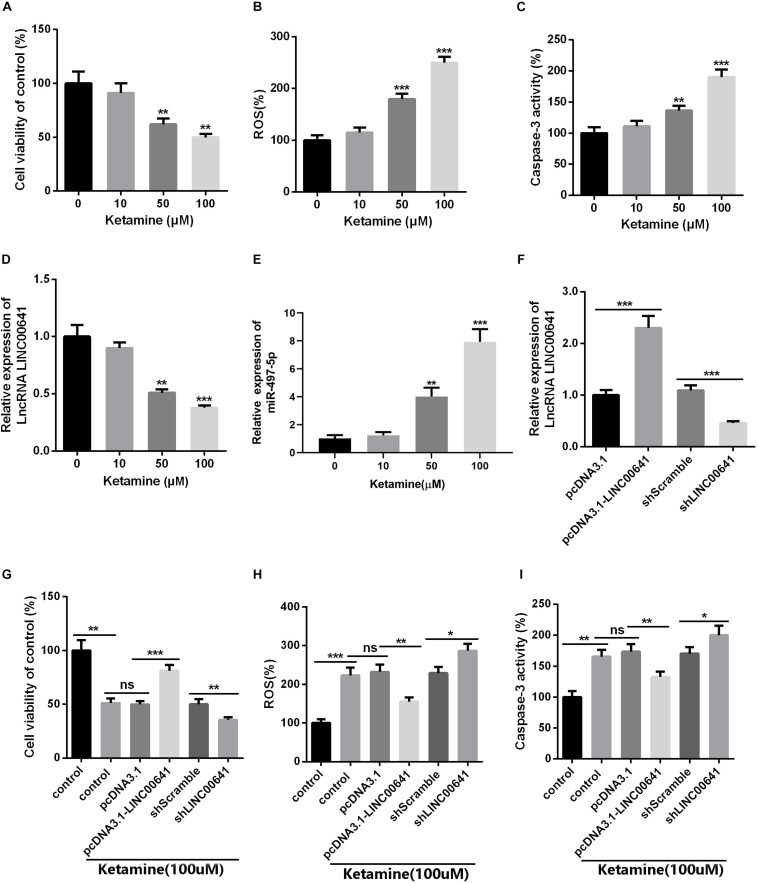
The effect of LINC00641 on ketamine-induced neuronal injury *in vitro*. Firstly, the neural stem cells treated with different concentrations of ketamine (0, 10, 50, and 100 μM) *in vitro*. **(A)** Cell viability was determined by MTT assay. **(B)** ROS levels in neural stem cell lines treated with different concentrations of ketamine. **(C)** caspase-3 activity was determined. **(D,E)** qRT-PCR was used to analyze the relative expression of LINC00641 and miR-497-5p in neural stem cell lines; **P < 0.01 and ***P < 0.001 compared with 0 mg/kg group. Then the neural stem cells were under 100 μM ketamine treatment and the shLINC00641 or pcDNA3.1. **(F)** The relative expression of LINC00641 in LINC00641 cell line (pcDNA3.1-LINC00641) and silencing cell line (shLINC00641) was analyzed by qRT-PCR. **(G–I)** The cell viability, ROS level and caspase-3 activity of neural stem cells in LINC00641 cell line and silencing cell line (shLINC00641) were measured. ns. *P* > 0.05, *P < 0.05, **P < 0.01, and ***P < 0.001.

To further investigate the effects of ketamine on neurons *in vitro*, rat brain embryonic stem cell-derived neuronal cells were used as cell model and treated with different concentrations of ketamine (0, 10, 50, and 100 μM). The cellular viability was detected by MTT, as the result revealed that neuronal cell viability was significantly reduced (Figure 2A), while ROS levels and caspase-3 activity were significantly increased (Figures 2B,C) under ketamine treatment compared to control group, indicating that neurons were severely damaged by high concentrations of ketamine. Moreover, qPCR results implied that ketamine dose-dependently repressed the LINC00641, while promoted the miR-497-5p expression (Figures 2D,E). To further verify the role of LINC00641 in ketamine-induced neuronal injury, we constructed LINC00641 overexpressing and low-expressing cell lines, respectively (Figure 2F). Further analysis of neuronal cell viability revealed that LINC00641 overexpression remarkably enhanced cell viability and suppressed ROS and caspase-3 levels compared to the 100 μM ketamine-treated group; whereas low expression of LINC00641 had the opposite effect (Figures 2G–I). These results indicate that LINC00641 overexpression can ameliorate ketamine-induced neurological injury to some extent.

### LINC00641 Targeted miR-497-5p

**FIGURE 3:**
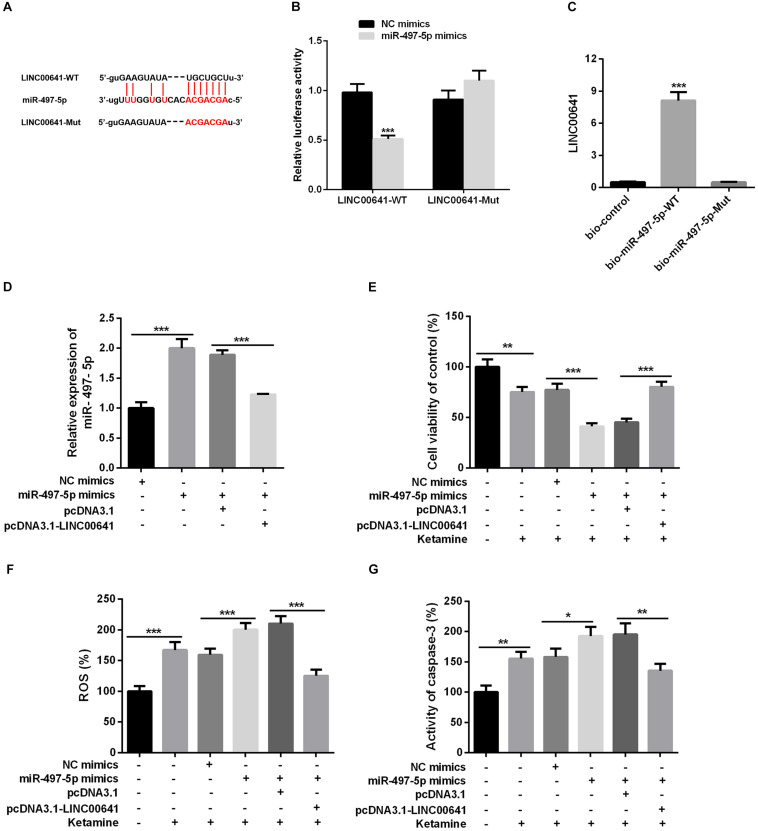
LINC00641 targeted miR-497-5p. **(A)** The binding sites between LINC00641 and miR-497-5p. **(B)** Dual-luciferase reporter assay was conducted to verify the relationship between miR-497-5p and LINC00641, ***P < 0.001 vs. NC mimic group. **(C)** RNA pull-down assay showed that miR-497-5p could directly bind LINC00641, **P < 0.01 vs. bio-control group. **(D)** qPCR was employed to detect the relative expression after overexpression of LINC00641 in miR-497-5p mimics cell line. **(E)** MTT assay was adopted to detect neuronal cell viability in different cell lines. **(F)** Neuronal cell ROS levels in different cell lines was detected by ROS detection kit. **(G)** Caspase-3 activity was detected by caspase3 detection kit. *P < 0.05, **P < 0.01, and ***P < 0.001.

To explore whether LINC00641 can interact with some miRNAs to improve the molecular mechanisms of ketamine-induced neuronal injury. The downstream miRNA of LncRNA was predicted and analyzed by miRNA prediction website StarBase^1^. The results showed that there were binding sites between LINC00641 and miR-497-5p (Figure 3A). To further verify the targeting relationship between them, we performed the dual-luciferase reporter assay and it showed that miR-497-5p could impede the luciferase activity of LINC00641-wt, but had no significant effect on the mutant form of LINC00641. At the same time, we conducted RNA pull-down assay to further validate whether LINC00641 could directly interact with miR-497-5p. These results indicated that LINC00641 could specifically bind to miR-497-5p (Figures 3B,C). Furthermore, we overexpressed LINC00641 in the miR-497-5p-overexpressed cell line, and found that the level of miR-497-5p was significantly repressed by LINC00641 overexpression (Figure 3D). Meanwhile, the cell viability was remarkably decreased by upregulating miR-497-5p, and the ROS level and caspase-3 activity were significantly increased (Figures 3E–G), indicating that miR-497-5p induced damages in neurons by aggravating the cytotoxic effects of ketamine. However, LINC00641 dampened those effects induced by miR-497-5p. Collectively, the above results demonstrated that LINC00641 could target miR-497-5p and ameliorates the ketamine-induced neuronal injury through inhibiting miR-497-5p.

### miR-497-5p Targeted Brain-Derived Neurotrophic Factor

**FIGURE 4:**
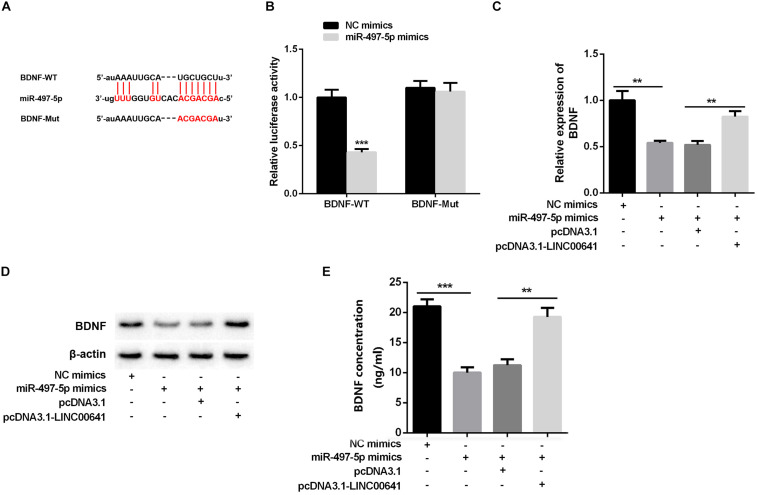
BDNF was inhibited by miR-497-5p. **(A)** The binding sites of BDNF and miR-497-5p were predicted through Targetscan (http://www.targetscan.org/vert_72/). **(B)** Dual-luciferase reporter assay was adopted to validate the binding relationship of miR-497-5p on the 3′ UTR sites of BDNF, ***P < 0.001 vs. NC mimic group. **(C)** qPCR was adopted to detect BDNF mRNA expression after overexpression of LINC00641 in miR-497-5p mimics cell line. **(D)** Western blot and **(E)** ELISA were employed to detect the protein expression of BDNF. **P < 0.01 and ***P < 0.001.

Studies have shown that miR-497-5p can bind to the 3′ UTR binding site of BDNF (Wang et al., 2017b; Li, 2018). First, we used the luciferase assay system to determine the effect of miR-497-5p on BDNF translation. Luciferase reporter assays revealed that miR-497-5p significantly suppressed the luciferase activity of BDNF-WT and had no significant effect on luciferase activity of BDNF-Mut (Figures 4A,B). Additionally, qPCR, Western Blot, and ELISA were carried out to test the BDNF expression under selectively regulation of LINC00641 and miR-497-5p. The results implied that the BDNF mRNA expression and protein were remarkably impeded in the miR-497-5p overexpressed cell line (vs. NC mimics). After pcDNA3.1-LINC00641 was transfected, the BDNF expression was significantly up-regulated (vs. miR-497-5p mimics + pcDNA3.1) (Figures 4C–E). These results indicated that miR-497-5p targeted at the 3′ UTR site of BDNF thus repressing its expression.

### LINC00641 Improved Ketamine-Induced Neuronal Injury by Activating TrkB/PI3K/Akt Signaling Pathway

**FIGURE 5:**
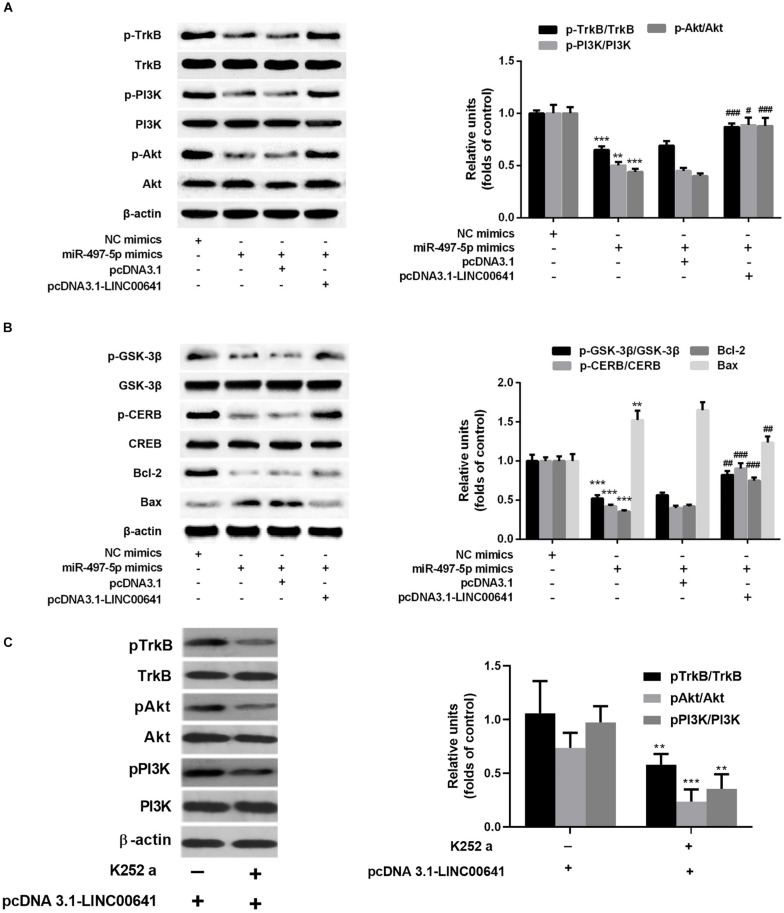
LINC00641 ameliorated ketamine-induced neurological injury by activating the TrkB/PI3K/Akt signaling pathway. **(A)** Western blot was used to detect the expression variable of p-TrkB, p-PI3K and p-Akt protein and the expression of relative protein after miR-497-5pmimics was added to LINC00641 cell line. **(B)** Protein expression and relative protein expression levels of apoptosis-related proteins p-GSK-3β, p-CERB, Bcl-2, and Bax in miR-497-5p mimics were added to the overexpressing LINC00641 cell line. **(C)** The LINC00641-overexpressed cell line was treated with TrkB inhibitor (K-252a, 1.7 nM), and Western blot was used to detect p-TrkB, p-PI3K, and p-Akt expression of relative protein. *P < 0.05, **P < 0.01, and ***P < 0.001 vs. NC mimics group. ^#^*P* < 0.05, ^##^*P* < 0.01, and ^###^*P* < 0.001 vs. miR-497-5p mimics + pcDNA3.1 group.

Previous studies have shown that BDNF can regulate cell growth via activating TrkB thereby activating the PI3K/Akt signaling pathway. Presently, to explore the downstream molecular mechanism of LINC00641/miR-497-5p/BDNF axis, we first examined the protein expression of TrkB/PI3K/Akt via Western blot. The results revealed that the p-TrkB, p-PI3K, and p-Akt expression was significantly down-regulated in the miR-497-5p mimics cell line (vs. pcDNA3.1). In contrast, the p-TrkB, p-PI3K and p-Akt expressions were significantly up-regulated in LINC00641-overexpressed cell line, which substantially attenuated the inhibition of p-TrkB, p-PI3K, and p-Akt induced by miR-497-5p (vs. pcDNA3.1-LINC00641 + NC mimics) (Figure 5A). It has recently been reported that activation of the PI3K/Akt/GSK-3β signal has a protective effect on ketamine-induced neuronal apoptosis (Beurel et al., 2011; Chan et al., 2012; Li et al., 2013). The phosphorylation level of GSK-3β also varied with p-Akt in this study (Figure 5B). The detection of a downstream transcriptional regulator CREB of Akt and the downstream pro-apoptotic protein Bax and anti-apoptotic protein Bcl-2 of CREB showed that CREB phosphorylation and Bcl2 expression were significantly decreased in miR-497-5p mimics cell line. The Bax expression was significantly increased (vs. pcDNA3.1), whereas LINC00641 overexpression remarkably attenuated this trend (vs. pcDNA3.1-LINC00641 + NC mimics) (Figure 5B). Additionally, the TrkB inhibitor could significantly reverse the p-TrkB, p-PI3K, and p-Akt up-regulated in LINC00641-overexpressed cell line (Figure 5C). Therefore, these results indicate that LINC00641 activates the TrkB/PI3K/Akt signaling pathway to ameliorate ketamine-induced neurological injury via modulating miR-497-5p/BDNF expression.

### To Verify the Effect of LINC00641 and PI3K Pathway on Ketamine-Induced Neuronal Injury in Rats

**FIGURE 6:**
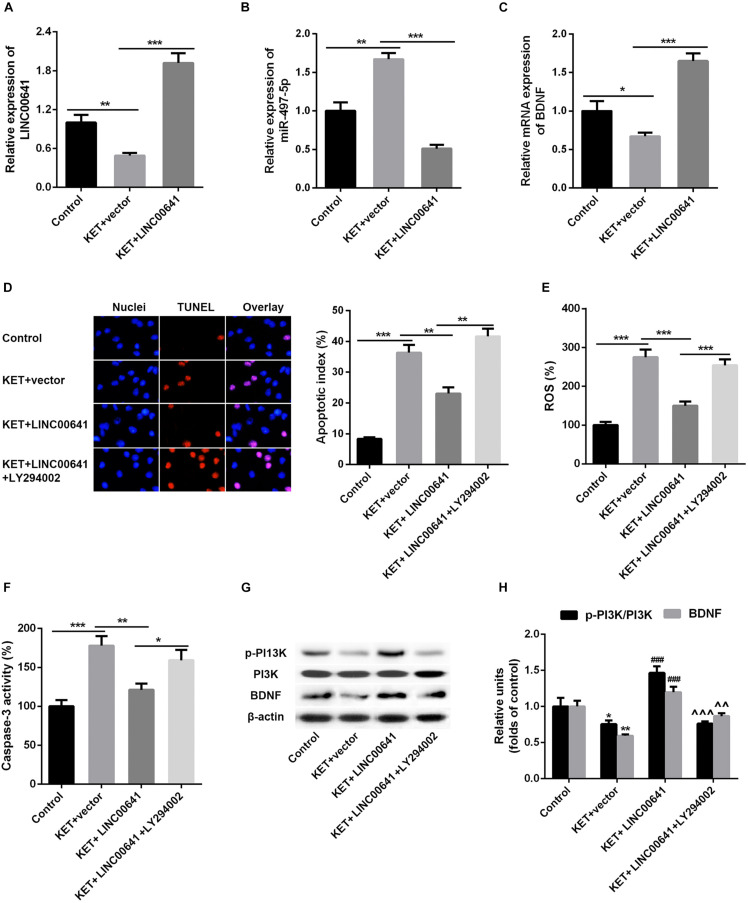
Effect of overexpression of LINC00641 on neuronal injury in rats. **(A–C)** The expression of miR-497-5p and BDNF mRNA in overexpressing LINC00641 rats was detected by qPCR. **(D–F)** TUNEL and apoptosis index, ROS level, caspase-3 activity analysis. **(G,H)** Western blot was used to detect the protein expression and relative protein expression of p-PI3K and BDNF. KET, ketamine. *P < 0.05, **P < 0.01, and ***P < 0.001 vs. control group. ^#^*P* < 0.05, ^##^*P* < 0.01, and ^###^*P* < 0.001 vs. KET + *vector group*. ^∧^*P* < 0.05, ^∧∧^*P* < 0.01, and ^∧∧∧^*P* < 0.001 vs. KET + LINC00641 group.

**FIGURE 7:**
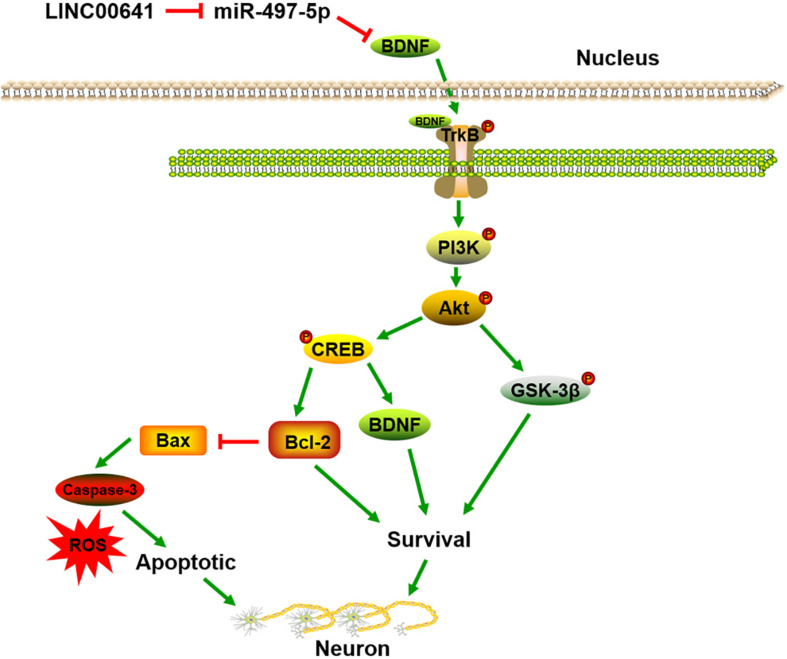
LINC00641/miRNA/BDNF pathway diagram for ameliorating ketamine-induced neuronal injury.

To further validate the effect of LINC00641 on ketamine-induced neuronal injury, we performed the stereotactic localization surgery in the hippocampus of rats. We injected adenovirus carrying the LINC00641 overexpression vectors in the hippocampus to overexpress LINC00641 in rat hippocampus cells (Figure 6A). The miR-497-5p expression in the brain cells of overexpressing LINC00641 rats was significantly down-regulated, and the mRNA expression level of BDNF was remarkably up-regulated (vs. control) (Figures 6B,C). TUNEL assay revealed that the apoptosis index of brain cells in the overexpressed rat brain of LINC00641 was significantly decreased, and the ROS level and caspase-3 activity were significantly increased (Figures 6D–F). To demonstrate the role of PI3K signaling pathway in LINC00641 mediated protective effects in ketamine-induced neuronal injury, we treated the rats with PI3K inhibitor LY294002. The results showed that the apoptosis index was significantly increased, the level of ROS and the activity of caspase-3 were significantly decreased (vs. KET + LINC00641) in the brain tissue treated with inhibitor LY294002 (Figures 6D–F). Additionally, the p-PI3K expression was significantly increased in the brains of rats with overexpressed LINC00641, and LY294002 significantly repressed the p-PI3K expression (Figures 6G,H). This indicates that LINC00641 activates the downstream PI3K signaling pathway via modulating the miR-497-5p/BDNF expression, thereby improving cell survival rate and ameliorating ketamine-induced neuronal injury (Figure 7).

## Discussion

Increasing evidences have revealed that high concentrations of ketamine could cause significant neuronal injury (Gutierrez et al., 2010; Turner et al., 2012). This study reports for the first time that LINC00641 exerts a protective effect on ketamine-induced neuronal damage. Mechanistically, LINC00641 can suppress the miR-497-5p expression by targeting it, facilitate the BDNF expression, and activate TrkB/PI3K/Akt signaling pathway to ameliorate ketamine-induced neuronal injury.

Although widely used as an anesthetic in the clinic, ketamine has been found to cause neuronal injury (Li et al., 2017). For instance, ketamine led to learning and memory impairment in adult rats when administered to the rats during brain development at a dose of 75 mg/kg (Huang et al., 2012). Besides, previous clinical studies with rodent and non-human primate models have shown that ketamine can induce accelerated programmed neuronal apoptosis at high doses and, or prolonged exposure of it (Wang F. et al., 2017). Here, our data revealed that the nerve cell viability was significantly decreased under the treatment of high concentration of ketamine (30 mg/kg body *in vivo* or 100 μM *in vitro*), and the ROS level and caspase-3 activity were significantly increased. Thus, the high concentration of ketamine can cause a certain degree of injury to neuronal cells.

In recent years, studies have found that LncRNA plays an important role in the development and function of the nervous system (Iyengar et al., 2014). For example, studies have shown that lncRNA-MEG3 overexpression can inhibit the PI3K/Akt pathway, increase the Bax, p53 and cleavage caspase-3 expression, and enhance neuronal injury induced by subarachnoid hemorrhage (SAH) (Liang et al., 2018). In addition, inhibition of lncRNA IGF2AS can protect neural stem cells from neurotoxicity induced by anesthetics via regulating the IGF2 expression and its related downstream signaling pathways (Song et al., 2017). As a member of lncRNAs, LINC00641 has been confirmed to play a role not only in the progression of multiple cancers including NSCLC (Li et al., 2019), breast cancer (Mao et al., 2020), and bladder cancer (Li et al., 2018), but also in intervertebral disk degeneration (Wang et al., 2019). Therefore, we are very curious about the role of LINC00641 in ketamine-induced neuronal injury. Our study demonstrated that the LINC00641 expression was significantly down-regulated under high concentration of ketamine treatment, while the LINC00641 overexpression *in vivo* and *in vitro* remarkably ameliorated the injury of ketamine on neuronal cells.

MicroRNAs-mediated regulation of gene expression is an important component of neural network regulation and is vital for neuronal differentiation and synapse formation. For instance, researches have shown that lsa-miR-375 impedes ketamine-induced neuronal cell death and neurotoxicity induced by lentiviral-mediated gene knockdown (Zhao et al., 2019). Furthermore, lncRNAs can function as competitive endogenous RNAs (ceRNAs) to spinge specific miRNAs through sponges, while miRNAs can specifically interfere the translation and protein expression with mRNA at the post-transcriptional level by specifically binding to the 3′ UTR end of target mRNAs (Cesana et al., 2011; Wang et al., 2017a). For instance, LINC00707 alleviates lipopolysaccharide-induced inflammation and apoptosis of PC-12 cells via targeting miR-30a-5p/Neurod (Zhu et al., 2019). The results of this study indicate that LINC00641 sponged miR-497-5p, which then targeted at the 3′ UTR site of BDNF. Thus, LINC00641 indirectly promoted BDNF expression through inhibiting miR-497-5p and then relieved the neuronal injury induced by ketamine.

Brain-derived neurotrophic factor is a member of the neurotrophin superfamily that enhances neuronal survival and growth (Park and Poo, 2013). Studies have shown that BDNF is a sort of neuroprotective secretory protein that binds extracellularly to the TrkB and activates the PI3K/Akt signaling pathway to enhance neuronal cell survival. The PI3K/Akt pathway plays a key role in neuronal survival and maintenance of diverse neuronal functions, and multiple apoptosis/survival regulatory molecules are downstream substrates of Akt, including CREB and GSK-3β (Frebel and Wiese, 2006; Zhang et al., 2013; Zhou et al., 2014). CREB is a transcription factor that promotes cell survival through transcriptional regulation. During transcriptional regulation, CREB up-regulates the expression of anti-apoptotic genes such as BDNF and Bcl-2. Moreover, Bcl-2 is an anti-apoptotic protein that represses apoptosis via preventing subsequent activation of Elease and caspase of cytochrome c (Unoki et al., 2016). In addition, Bax is a proapoptotic protein downstream of Bcl-2 (Suzanne et al., 2003). GSK-3β is another major downstream protein of Akt and is critical for PI3K-mediated neuronal survival (Hetman et al., 2000). Previous studies have revealed that ketamine induces neuronal apoptosis by down-regulating the Bcl-2 expression (Huang et al., 2012). In this research, ketamine reduced the p-TrkB, p-PI3K and p-Akt expression in neuronal cells, inhibited the activity of GSK-3β and CREB, down-regulated Bcl-2 expression, and activity of Bax and caspase-3 was significantly increased. Additionally, LINC00641 overexpression in the PC12 cell line significantly attenuated the inhibition of the PI3K/Akt pathway induced by transfection of miR-497-5p mimics. To further verify whether LINC00641 regulates neuronal apoptosis through this pathway, we injected PI3K inhibitor LY294002 in overexpressing LINC0064 large rats. The results showed that the addition of inhibitor LY294002 remarkably attenuated the improved effect of LINC0064 overexpression on PI3K/Akt activation and ketamine-induced neuronal injury. These results indicate that LINC00641 may activate the TrkB/PI3K/Akt pathway via miR-497-5p/BDNF to protect nerve cells injured by ketamine (Figure 7).

In summary, we demonstrate that LINC00641 ameliorated ketamine-induced neuronal injury. Functionally, it acts as an endogenous competitive RNA via sponging miR-497-5p, thereby indirectly promoting BDNF expression and activating TrkB/PI3K/Akt signaling pathway. Therefore, the regulation of the LINC00641 expression is expected to be a new approach to alleviate ketamine-induced neuronal injury.

## Data Availability Statement

The raw data supporting the conclusions of this article will be made available by the authors, without undue reservation, to any qualified researcher.

## Ethics Statement

The animal study was reviewed and approved by The First Hospital of Quanzhou Affiliated of Fujian Medical University.

## Author Contributions

QC and WqX: manuscript design and writing. QC, WjX, and ML: experimental design and data analysis. QC, JY, and YY: data analysis and figures. All authors read and approved the final manuscript.

## Conflict of Interest

The authors declare that the research was conducted in the absence of any commercial or financial relationships that could be construed as a potential conflict of interest.

## Footnotes

1 http://starbase.sysu.edu.cn/agoClipRNA.php?source=lncRNA
